# kGCN: a graph-based deep learning framework for chemical structures

**DOI:** 10.1186/s13321-020-00435-6

**Published:** 2020-05-12

**Authors:** Ryosuke Kojima, Shoichi Ishida, Masateru Ohta, Hiroaki Iwata, Teruki Honma, Yasushi Okuno

**Affiliations:** 1 Graduate School of Medicine, Kyoto University, Shogoin-kawaharacho, Sakyo-ku, Kyoto, 606-8507 Japan; 2 Graduate School of Pharmaceutical Sciences, Kyoto University, Yoshida, Sakyo-ku, Kyoto, 606-8501 Japan; 3 Medical Sciences Innovation Hub Program, RIKEN Cluster for Science, Technology and Innovation Hub, Tsurumi-ku, Kanagawa, Kanagawa, 230-0045 Japan; 4 RIKEN Center for Biosystems Dynamics Research, Tsurumi-ku, Kanagawa, Kanagawa, 230-0045 Japan

**Keywords:** Graph convolutional network, kGCN, Graph neural network, Open source software, KNIME

## Abstract

Deep learning is developing as an important technology to perform various tasks in cheminformatics. In particular, graph convolutional neural networks (GCNs) have been reported to perform well in many types of prediction tasks related to molecules. Although GCN exhibits considerable potential in various applications, appropriate utilization of this resource for obtaining reasonable and reliable prediction results requires thorough understanding of GCN and programming. To leverage the power of GCN to benefit various users from chemists to cheminformaticians, an open-source GCN tool, kGCN, is introduced. To support the users with various levels of programming skills, kGCN includes three interfaces: a graphical user interface (GUI) employing KNIME for users with limited programming skills such as chemists, as well as command-line and Python library interfaces for users with advanced programming skills such as cheminformaticians. To support the three steps required for building a prediction model, i.e., pre-processing, model tuning, and interpretation of results, kGCN includes functions of typical pre-processing, Bayesian optimization for automatic model tuning, and visualization of the atomic contribution to prediction for interpretation of results. kGCN supports three types of approaches, single-task, multi-task, and multi-modal predictions. The prediction of compound-protein interaction for four matrixmetalloproteases, MMP-3, -9, -12 and -13, in the inhibition assays is performed as a representative case study using kGCN. Additionally, kGCN provides the visualization of atomic contributions to the prediction. Such visualization is useful for the validation of the prediction models and the design of molecules based on the prediction model, realizing “explainable AI” for understanding the factors affecting AI prediction. kGCN is available at https://github.com/clinfo.

## Introduction

Deep learning is emerging as an important technology to perform various tasks in cheminformatics [1–3]. With the recent development of artificial intelligence (AI) and deep learning, the application of deep learning approaches has been practically demonstrated for various predictions such as virtual screening [4], quantitative structure-activity relationship (QSAR) studies [5], and ADMET (absorption, distribution, metabolism elimination, and toxicology) prediction [6, 7]. In particular, with the democratization of AI, it is expected that these prediction tools should be readily used by the non-experts. The accessibility of deep learning to non-experts is an important issue in the field of cheminformatics. For example, as deep learning can be applied to a wide range of research areas in drug discovery such as ADMET predictions for lead optimization and virtual screening for lead identification, the chemists should be able to solve these research problems by using the latest technologies and analyze the results, availing the benefits of deep learning. However, as chemists are typically not proficient in deep learning, the development of easy-to-use, multi-functional deep learning software is necessary.

In the predictions based on molecular structures, graph neural networks (GNNs), where a chemical structure is represented as a graph, have been reported to perform well [8, 9]. In particular, graph convolutional networks (GCNs), a type of GNN, exhibited excellent performances in many applications [10, 11]. Despite these results, an appropriate application of GCN to real-world research problems requires practical programming skills and comprehensive understanding of deep learning and GCN.

To address this issue, a new open-source software, kGCN^1^. is introduced for various users to employ deep learning including GCNs. kGCN is developed for the following functions:

- Providing interfaces for the various levels of users including users with limited programming skills
- Handling different types of data for cheminformatics tasks
- Easy, intuitive, and convincing interpretation of results
- Hyper-parameter optimization

**Fig. 1:**
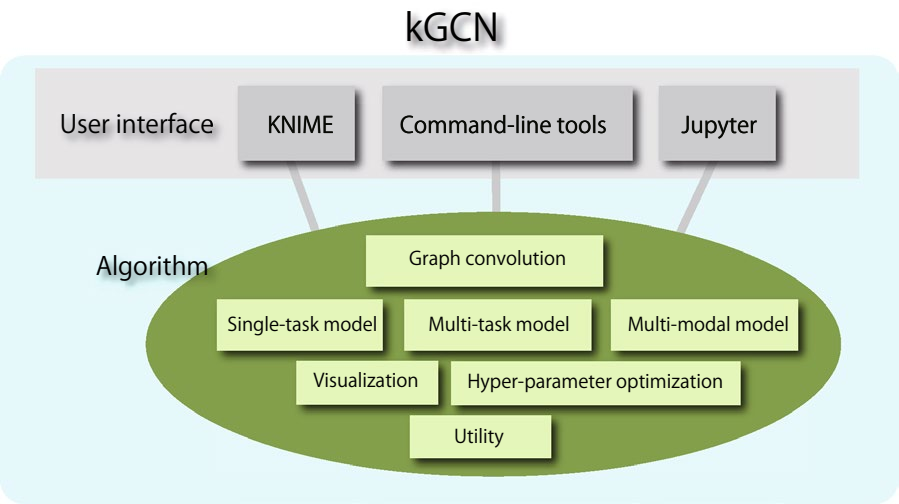
Architecture of kGCN

As mentioned, one function of kGCN is to afford interfaces to assist various users such as chemists, cheminformaticians, and data scientists. Considering the expertise levels of these users, a software should provide multiple interfaces suitable for each user. To satisfy these requirements, kGCN provides three types of user interfaces. Figure 1 shows the architecture of the kGCN system. The kGCN system supports both GUI-based and command-line interfaces. To intuitively access a machine-learning procedure, the kGCN system provides a GUI interface on the GUI platform, KNIME (Konstanz Information Miner) [12]. The command-line interface supports typical machine-learning procedures such as training, evaluation, and cross-validation. Additionally, the kGCN modules can be used as a Python library to allow flexibility and processing through programming languages.

The second function is to support different types of data. In cheminformatics, various types of data including chemical structures represented by graphs shoulded be considered. For example, the protein sequence data is often represented as a symbol sequence or vector descriptor. In deep learning, various architectures for neural networks have been proposed [13]. The simplest GCN is based on the single-graph-input single-label-output architecture. The kGCN system supports 1) multi-input (multi-modal GCN) and 2) multi-output (multi-task GCN) architectures. A multi-modal GCN is a neural network that can accept multiple modalities of inputs [14, 15]. kGCN can accommodate a neural network with two inputs: chemical structure as a graph and a protein sequence as a series of characters. This type of neural network can be used to predict interactions between the compounds and proteins for virtual screening and/or drug-repurposing [4, 16]. However, multiple related tasks are needed to be simultaneously handled in cheminformatics [17], for example, tasks to predict multiple different properties of a compound. To tackle these, a multi-task neural network is applied, which affords better results than those for an individual prediction [18, 19].

The third function is the interpretation and understanding of the cause of prediction results via deep learning by visualizing contributions of input data to the prediction. This process is important because the validity of the prediction model can be examined through a visual inspection of the good and bad features. The refinement or re-construction of the prediction model can be performed if the causes of prediction do not appear to be reasonable or are contrary to common sense. Notably, designing new molecules with improved properties is possible if the reasons for good and/or bad predictions are identified by visualization. In recent years, several methods to calculate the different contributions to the prediction results of deep learning have been proposed [20, 21]. The kGCN system uses the integrated gradient method [22], which can be applied to any type of neural network architectures including multi-task and multi-modal neural networks.

The last function is hyper-parameter optimization. In analysis using deep neural networks, hyper-parameters of deep learning such as the number of network layers, number of layer nodes, learning rate, and batch size should be appropriately set. However, setting these parameters is not easy for users without deep learning knowledge and experience. To assist such users and automatically determine the optimal hyper-parameters, the kGCN system employs Bayesian optimization and metaheuristics for hyper-parameter optimization [23].

In addition to this information, the kGCN system also provides tools for improving the usability. The kGCN back-end implementation uses Tensorflow [24] and supports GPUs (graphics processing units). To setup the execution conditions, kGCN-installed Docker images are also provided^2^. Additional unique tools to enhance the usability are provided for each interface. These will be described in the Implementation section.

Similar types of software have been reported in prior studies, e.g., DeepChem [25], Chainer chemistry [26], and OpenChem [27]. DeepChem is a Python library for neural networks, including GCNs. A notable feature of DeepChem is to support various machine learning methods as well as deep learning methods. Because deep learning usually requires large amounts of data, this feature can help users handle relatively small amounts of data. Chainer chemistry provides GCNs as an extended Python library of Chainer [28]. Both libraries can be used with Python and were developed for professional programmers of machine learning and Python. Although OpenChem supports both command-line and Python interfaces, good programming skills are still required to use OpenChem. The kGCN system is a framework containing the GUI, command-line, and Python interfaces. The GUI interface of kGCN is expected to engage users with limited programming skills in GCN and deep learning. To our knowledge, kGCN is the first open-source and multi-functional GCN software to support all three interfaces.

## Implementation

Before describing the details of the kGCN system, basic implementation techniques for the graph representation of molecules and graph convolution are discussed.

### Graph representation of molecules for GCN

This section first describes the formalization of a molecule to apply the GCNs. A molecule is formalized as a tuple $\mathcal{M} \equiv (V,E,F)$, where *V* is a set of nodes. A node represents an atom in a molecule. A node has features $\mathbf {f}_i \in F (i \in V)$, and *F* is a set of feature vectors representing the atom properties such as atom type, formal charge, and hybridization. These features should be appropriately designed by users. *E* is a set of edges, and an edge $e \in E$ represents a bond between the atoms, i.e., $e \in V \times V \times T$, where *T* is a set of bond types. An adjacency matrix $\mathbf {A}^{(t)}$ is used, which is defined as follows:

$$\begin{aligned} (\mathbf {A}^{(t)})_{i,j} = {\left\{ \begin{array}{ll} 1 &{} (v_i,v_j,t) \in E \\ 0 &{} (v_i,v_j,t) \notin E \end{array}\right. }, \end{aligned}$$

where $(\cdot )_{i,j}$ represents the *j*-th element of *i*-th row. Similarly, the feature matrix is defined as:

$$\begin{aligned} (\mathbf {F})_{j,k}=(\mathbf {f}_j)_k \end{aligned}$$

where $(\cdot )_{k}$ represents the *k*-th element of a vector.

Using this matrix, a molecule is represented by $\mathcal{M'} = (\mathbf {A},\mathbf {F})$, where $\mathbf {A} = \{\mathbf {A}^{(t)} | t \in T\}$. The framework in the present system uses RDKit [29] to create adjacency and feature matrices and employs $\mathcal M'$ as the input for GCN.

### Graph convolutional network

kGCN supports GCNs in addition to the standard feed-forward neural networks. Therefore, GCNs for molecules are described first. Graph convolution layer, graph dense layer, and graph gather layer are defined as described below.

#### Graph convolution layer

The graph convolution is calculated from the input $\mathbf {X^{(\ell )}}$ of the $\ell$-th layer as follows:

$$\begin{aligned} \mathbf {X}^{(\ell +1)} = \sigma \left( \sum _t \tilde{\mathbf {A}}^{(t)} \mathbf {X}^{(\ell )} \mathbf {W}^{(\ell )}_t \right) , \end{aligned}$$

where $\mathbf {X}^{(\ell )}$ is the $N \times D^{(\ell )}$ matrix and $\mathbf {W}^{(\ell )}_t$ is the parameter matrix ($D^{(\ell )} \times D^{(\ell +1)}$) for a bond type *t*, $\sigma$ is the activation function, and $\tilde{\mathbf {A}}^{(t)}$ is the normalized adjacency matrix ($N \times N$). This normalization and implementation of the layers follows Kipf’s model [30] as a default. There are various choices for implementing the setting of graph convolution layers. In the kGCN system, the operation of the first layer input can be easily switched by changing the initial setting file for building the model.

The GCN is based on this graph convolution operation. The input of the first layer $\mathbf {X}^{(1)}$ often corresponds to the feature matrix, $\mathbf {F}$

#### Graph dense layer

$\mathbf {X^{(\ell )}}$ is an input for graph dense layer. $\mathbf {X^{(\ell +1)}}$ is calculated as follows:

$$\begin{aligned} \mathbf {X}^{\ell +1} = \mathbf {X}^{(\ell )} \mathbf {W}^{(\ell )}, \end{aligned}$$

where $\mathbf {X}^{(\ell )}$ is an $N \times D^{(\ell )}$ matrix and $\mathbf {W}^{(\ell )}$ is a parameter matrix ($D^{(\ell )} \times D^{(\ell +1)}$).

#### Graph gather layer

This layer converts a graph into a vector [31], i.e., the input $\mathbf {X}^{(\ell )}$ is an $N \times D^{(\ell )}$ matrix and $\mathbf {X}^{(\ell )}$, i.e.,

$$\begin{aligned} (\mathbf {X}^{(\ell +1)})_{j}=\sum _j (\mathbf {X}^{(\ell )})_{ij}, \end{aligned}$$

where $(\cdot )_{i}$ represents an *i*-th element of a vector. This operation converts a matrix into a vector.

**Fig. 2:**
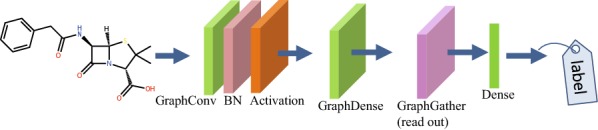
Graph convolutional network for a prediction task with a compound input

Figure 2 shows an example of GCN for a prediction task. The GCN model is a neural network consisting of a graph convolutional layer (GraphConv) with batch normalization (BN) [32] and rectified linear unit (ReLU) activation, graph dense layer with the ReLU activation, graph gather layer, and dense layer with the softmax activation. By assigning the label that is suitable for each task to the compounds, this model can be applied to many types of tasks, e.g., ADMET prediction based on the chemical structures.

**Fig. 3:**
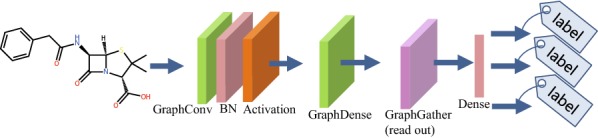
Multi-task graph convolutional network with a compound input

Figure 3 shows an example of a multi-task GCN for a prediction task. The only difference is that multiple labels are predicted as an output. In this type of neural networks, multiple labels associated with a molecule such as several types of ADMET properties can be predicted simultaneously. It is well-known that multi-task prediction affords more improvement in the performance compared to that of individual single-task prediction [33].

**Fig. 4:**
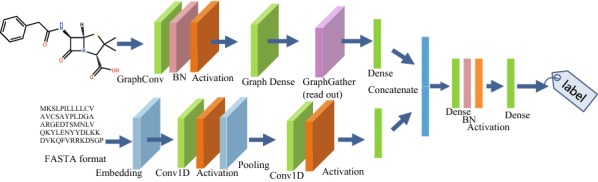
Multi-modal graph convolutional network with compound and sequence inputs

Figure 4 shows an example of a multi-modal neural network employing a graph representing a compound and sequence of a protein. In addition to the information derived from the molecular structure, information from other modalities can also be used for the input. An example of the prediction of activity using compound and protein related information is described in detail in the Experiment section.

The kGCN system supports operations described above and some other additional operations to build a neural network. These operations are implemented using TensorFlow [34] and are compatible with Keras [35], allowing the users to construct neural networks such as convolutional neural networks and recurrent neural networks [13] with Keras operations.

These neural networks include hyper-parameters such as the number of layers in a model and number of dimensions for each layer. To determine these hyper-parameters, the kGCN system includes Bayesian optimization.

### Visualization of graph convolutional network

To confirm the features of the molecules that influence prediction result, a visualization system using the integrated gradient (IG) method [22] is developed. After the construction of the prediction model, the visualization of the atom importance in the molecular structure, based on the IG value $\mathcal{I}(x)$ derived from the prediction model, is possible.

IG value $\mathcal{I}(x)$ is defined as follows:

$$\begin{aligned} \mathcal{I}(x) = \frac{x}{M}\sum _{k=1}^M \nabla S\left(\frac{k}{M}x\right), \end{aligned}$$

where *x* is the input of an atom of a molecule, *M* is the number of divisions of the input, *S*(*x*) is the prediction score, i.e., the neural network output with input *x*, and $\nabla S(x)$ is the gradient of *S*(*x*) related to input *x*. In the default setting, *M* is set to 100. The atom importance is defined as the sum of the IG values of features in each atom. The calculation of the atom importance is performed on compound-by-compound basis.

The evaluation of the visualization results depends on each case. Although methods for the visualization of deep learning results are still developing, their effectiveness in solving common problems has not been reported; however, a quantitative evaluation of the IG values related to the molecules was previously reported for the prediction of a reaction [36].

### Hyper-parameter optimization

To optimize the neural network models, hyper-parameters such as the number of graph convolution layers, the number of dense layers, dropout rate, and learning rate should be determined. As it is difficult to manually determine all these hyper-parameters, kGCN allows automatic hyper-parameter optimization with Gaussian-process-based Bayesian optimization using a Python library, GPyOpt [37].

### Interfaces

This section describes three interfaces in the kGCN system.

#### Command-line interface

The kGCN system provides the command-line interface suitable for batch execution. Data processing is designed according to the aim, but there is a standard process common to many data processing designs, e.g., a series of processes for cross-validation. The kGCN commands include these common processes, i.e., the kGCN system allows preprocessing, learning, prediction, cross-validation, and Bayesian optimization using the following commands:

**kgcn-chem command**: allows preprocessing of molecule data, e.g., structure-data file (SDF) and SMILES.
**kgcn command**: allows batch execution related to prediction tasks: supervised training, prediction, cross-validation, and visualization.
**kgcn-opt command**: allows batch execution related to hyper-parameter optimization.

These commands can be used with Linux commands and enable users to construct automatic scripts, e.g., Bash scripts. Because such batch execution is suitable for large-scale experiments using workstation and reproducible experiments, this interface is useful for the evaluation of neural network models.

#### KNIME interface

**Fig. 5:**
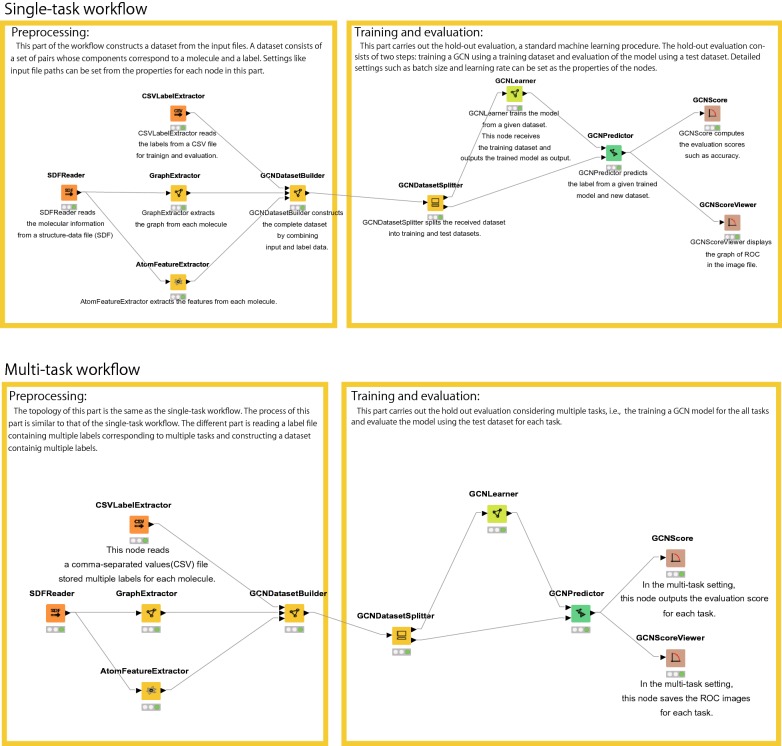
Single-task workflow for the hold-out procedure using the KNIME interface (Upper). Multi-task workflow for the hold-out procedure (Lower)

**Fig. 6:**
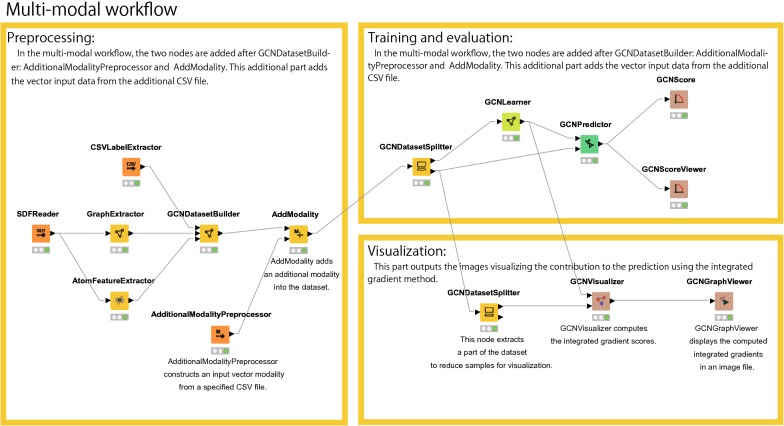
Multi-modal workflow for the hold-out procedure

The kGCN system supports KNIME modules as a GUI. KNIME is a platform to prepare the workflow, which consists of KNIME nodes for data processing, and is particularly useful in the field of data science. The kGCN KNIME nodes described below are useful for the execution of various kGCN functions in combination with existing KNIME nodes. The command-line interface allows batch execution, whereas the KNIME interface is suitable for early steps in the machine learning process such as prototyping and data preparation.

To train and evaluate the model, kGCN provides the following two nodes.

**GCNLearner**: trains the model from a given dataset. This node receives the training dataset and provides the trained model as an output. Detailed settings such as batch size and learning rate can be set as the node properties.
**GCNPredictor**: predicts the label from a given trained model and new dataset.

Using the kGCN nodes mentioned above, Fig. 5 shows an example of the workflow. This data flow can be separated into that before and after GCNLearner. The former part is for data preparation, for which kGCN includes the following KNIME nodes:

**CSVLabelExtractor**: reads labels from a CSV file for training and evaluation
**SDFReader**: reads the molecular information from an SDF.
**GraphExtractor**: extracts the graph from each molecule.
**AtomFeatureExtractor**: extracts the features from each molecule.
**GCNDatasetBuilder**: constructs the complete dataset by combining input and label data.
**GCNDatasetSplitter**: splits the dataset into training and test datasets.

The test dataset is used for the evaluation and interpretation of results. kGCN also provides the modules to display the output of the results.

**GCNScore**: provides the scores of the prediction model such as accuracy.
**GCNScoreViewer**: displays the graph of ROC scores in the image file.
**GCNVisualizer**: computes the IG values and atom importance.
**GCNGraphViewer**: displays the atom importance in the image file.

Another example of the workflow is shown in Fig. 6, which includes an example of multi-modal neural networks. To design multi-modal neural networks, the kGCN system provides the following modules:

**AdditionalModalityPreprocessor**: reads the data of another modality from a given file.
**AddModality**: adds the data of another modality to the dataset.

To change from single-task to multi-modal, AddModality node should be added next to the GCNDatasetBuilder node.

The visualization process shown at the bottom-right of Fig. 6 requires a specific computation time depending on the number of molecules to be visualized, as the computation time for the integrated gradient method for each molecule is 1–5 s during GPU execution. To reduce the size of the dataset, GCNDatasetSplitter can be used for selecting a part of the dataset.

#### Python interface

The kGCN system also provides a Python library for programmers to more precisely tune the setting of the analysis. The kGCN system can be used in a manner similar to any standard library and supports pip, a Python standard package manager. Furthermore, the kGCN system can be used in the Jupyter notebook, which is an interactive interface. Therefore, the users can easily explore this library using google collaboratory, a cloud environment for the execution of Python programs.

The kGCN system adopts an interface similar to scikit-learn, a defacto standard machine learning library in Python. Therefore, the process employing the kGCN library includes preprocessing, training by *fit* methods, and evaluation by *pred* method, in this order. The users can easily access the kGCN library in a similar manner to that of scikit-learn. Furthermore, designing a neural network, which is necessary for using kGCN, is easy if users are familiar with Keras because kGCN is compatible with the Keras library, and the users can easily design a neural network such as Keras.

To demonstrate a wide applicability of the present framework, three sample programs comprising the datasets and scripts using the standard functions of kGCN are available in the framework web pages. In addition to these examples, the application of kGCN for a reaction prediction has been reported in a prior study [36], where the visualized reaction centers predicted by GCNs were consistent with reaction centers reported in the literature. This literature report used GCNs for reaction prediction on the kGCN system.

#### Flexible user interfaces

As described in the introduction and implementation sections, kGCN provides KNIME GUI, a command-line interface, and a programming interface to support various types of users with various skill levels. For example, an easy-to-use high-layer GUI can assist the chemists with limited programming knowledge in using kGCN and understand SAR at a molecular level. Contrarily, for machine learning professionals with good programming skills, it is expected that they will focus on the improvement of algorithms using a low-layer python interface. By using a Python interface, the users can make machine learning procedures more flexible and incorporate the kGCN functions into the user specific programs such as web services. The users with good programming skills can also use the command-line interface to automate data-analysis procedures using the kGCN functions because it is easy to construct a pipeline combined with other commands such as Linux commands.

## Results

For applications of kGCN, this section describes the prediction of the assay results of a protein based on the molecular structure. The prediction of compound-protein interactions (CPIs) has played an important role in drug discovery [38], and CPI prediction methods using deep learning have achieved excellent results [4, 14–16]. In this study, the applicability of kGCN to CPI prediction is demonstrated as an example of single-task/multi-task/multi-modal GCNs. The single-task GCN predicts the activity against a protein based on the chemical structure represented as a graph. The multi-task GCN predicts the activities against multiple proteins from a chemical structure. Although single-task and multi-task GCNs do not use the information related to proteins, multi-modal neural networks predict the activity from information of both the protein sequence and chemical structure.

For this examination, a dataset was prepared from the ChEMBL ver.20 database. The threshold for active/inactive was defined as 30uM. This dataset consists of four types of matrix metalloprotease inhibition assays, MMP-3, MMP-9, MMP-12, and MMP-13. The number of compounds for each assay are listed in Table 1. These MMPs were selected because relatively large amounts of data were available for these in the ChEMBL dataset [39].

**Table 1:** Number of compounds in our dataset

Assay type	$\#$Compounds
MMP-3	2095
MMP-9	2829
MMP-12	533
MMP-13	2607

kGCN provides many types of descriptors for a compound and protein. For example, kGCN allows graph representation for GCN and vector representation, such as ECFP [40] and DRAGON [41], for standard neural networks. Additionally, to represent a protein, kGCN uses an amino-acid sequence and vector representation such as PROFEAT descriptors [42]. This application uses graph representation for a compound and sequence representation for a sequence.

To simplify the experiment, the molecules with greater than 50 atoms were removed. As the dataset was unbalanced, negative data corresponding to inactivity were selected in the same manner [14]. Negative data was generated to equalize the number of negative and positive data for each assay.

Such preprocessing can be realized using the kgcn-chem command included in the section describing the command-line interface.

**Fig. 7:**
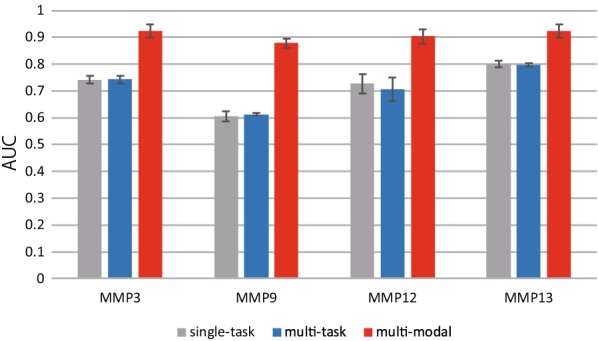
AUCs obtained from five-fold cross-validation

**Fig. 8:**
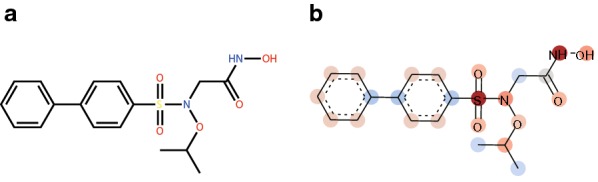
**a** Chemical structure.** b** Atomic contributions to the predicted MMP-9 activity. Red color represents the positive contribution to the prediction (MMP-9 active in this case). Blue color represents the negative contribution (not active)

Figure 7 shows the area under the curve in the receiver operator characteristic curve (ROC-AUC) of five-fold cross-validation. This result shows that the multi-modal approach outperforms the other approaches. The reason for a better ROC-AUC of the prediction with multi-modal approach is speculated to be the use of sequence-related information of the target proteins in addition to the graph representation of the compounds. This result is consistent with the reported results which indicate that the sequence descriptor contributes to improved accuracy [4, 14–16].

kGCN allows the visualization of the atomic contributions to the prediction result, as shown in Fig. 8b. The compound, N-hydroxy-2-[N-(propan-2-yloxy)[1,1’-biphenyl]-4-sulfonamido]acetamide (Fig. 8a), is used for this prediction and its reported activity 200 nM (IC50) against MMP-9 [43]. The label of this compound for MMP-9 in the dataset is active, and the activity predicted for this compound in single-task mode is correct (probability of active label is 0.964). This compound possesses a hydroxamic acid group (-C(=O)NHOH), and it is well-known that many MMP inhibitors have a hydroxamic group. The crystallographic structure of a complex of MMP-9 and this compound has been previously reported [44]. MMP-9 is a zinc protease, and the hydroxamic acid group of the above compound is coordinated to the zinc ion of MMP-9. The positive contributions of OH, NH, and carbonyl oxygen of the hydroxamic acid group shown in Fig. 8b are consistent with the interaction of the hydroxamic group with zinc of MMP-9.

Such visualization can be used to confirm the validity of the prediction by comparing the atomic contributions toward the prediction with structure-activity and/or -property relationships. Additionally, this visualization can be useful for drug designing to improve the activity, physicochemical properties and/or ADMET properties by modifying the chemical moieties that contribute negatively to the prediction.

## Conclusion

For assisting various users including chemists and cheminformaticians, an open-source GCN tool, kGCN, is described. To support the users with various levels of programming skills, kGCN provides three interfaces: a GUI using the KNIME platform for users with limited programming skills such as chemists, as well as command-line and Python library interfaces for the advanced users such as cheminformaticians and data scientists. Three steps including preprocessing, model tuning, and interpretation of results, required for building a prediction model and utilization of prediction results. kGCN supports these three steps by including functions such as the automatic preparation of graph representation based on the chemical structures for pre-processing, Bayesian optimization for automatic optimization of the hyper-parameters of the neural networks for model tuning, the integrated gradient method to visualize the atomic contribution toward the prediction result for interpretation. In terms of the approaches used for prediction, kGCN supports single-task, multi-task, and multi-modal predictions. The CPI prediction for four assays of matrixmetalloprotease inhibition, MMP-3, -9, -12, and -13, is performed as a representative case study using kGCN. Multi-modal prediction shows higher accuracy than those of the single-task and multi-task predictions. Additionally, the visualization of atomic contribution to the prediction indicated that hydroxamate group of the compound exhibits a positive contribution to the activity and this is consistent with the known structure-activity relationships. Such visualization is useful for the validation of the models and designing new molecules based on the model. This also allows the realization of “explainable AI” for understanding the factors influencing the AI prediction which are typically a black-box.

kGCN is available at https://github.com/clinfo/kGCN. Various examples such as Jupyter notebooks are also provided. Future works will include supporting new methods of graph neural networks because graph neural networks are a hot topic at present and new methods, e.g., graph attention and pooling, are being actively developed. We will proactively adopt these new methods and continue to develop kGCN so that various users can easily apply such latest methods to appropriately analyze the data in their hands and understand the reasons for the predictions. Also, we are going to gather the user feedback and improve kGCN for better usability.

## Data Availability

Project name: kGCN. Project home page: https://github.com/clinfo/kGCN. Operating system(s): Platform independent(Ubuntu 18.04, and CentOS 7 are mainly supported). Programming language: Python. Other requirements: python3 (> 3.6), tensorflow. License: https://github.com/clinfo/kGCN/blob/master/LICENSE. Any restrictions to use by non-academics: licence needed.

## Footnotes

1 Kyoto-university graph convolutional network framework.

2 https://hub.docker.com/r/clinfo/kgcn.
